# Impact of alfalfa (*Medicago sativa* L.) galactomannan on the microstructural and physicochemical changes of milk proteins under static *in-vitro* digestion conditions

**DOI:** 10.1016/j.fochx.2022.100330

**Published:** 2022-05-18

**Authors:** Thierry Hellebois, Claire Gaiani, Christos Soukoulis

**Affiliations:** a Environmental Research and Innovation (ERIN) Department, Luxembourg Institute of Science and Technology (LIST), 5 avenue des Hauts-Fourneaux, Esch-sur-Alzette, L4362, Luxembourg; b Université de Lorraine, LIBio, Nancy, France; c Institut Universitaire de France (IUF), France

**Keywords:** In-vitro digestion, Proteolysis, Lucerne, Sodium caseinate, Whey protein, Colloidal changes

## Abstract

This paper reports on the impact of alfalfa galactomannan (AAG, 0.1, 0.5 or 1% wt.) on the colloidal changes and digestibility of sodium caseinate (NaCN) and whey protein isolate (WPI) dispersions (10% wt.) under static *in-vitro* digestion conditions. Static laser light scattering and confocal laser scanning microscopy-assisted assessment of the NaCN-based gastric chymes confirmed the ability of AAG to control the acid-induced protein coagulation phenomena. Contrarily, the presence of AAG in the WPI-based gastric chymes was associated with the formation of larger aggregates due to the occurrence of segregative microphase separation. The kinetic modelling of the SDS-PAGE densitometric data showed that the intragastric peptic cleavage rates were higher for caseins than whey proteins (β-lactoglobulin, α-lactalbumin). However, free amino acid (FAA) release rates did not exceed 12% under intragastric conditions, whilst notably higher release rates were achieved in the intestinal digesta (36–52%). In all cases, the FAA release rates significantly increased in the presence of AAG.

## Highlights

• The impact of alfalfa galactomannan (AAG) on the digestibility of milk proteins was studied.
• AAG mediated the intragastric aggregation of both sodium caseinate and whey protein isolate.
• AAG affected only the peptic cleavage of caseins and β-lactoglobulin in the gastric chymes.
• AAG enhanced the free amino acids release in the gastric chymes regardless of the protein type.
• The free amino acids release rate in the intestinal chymes were adversely related to AAG content.

## Introduction

Milk proteins constitute food ingredients with high nutritional value and biological activity, while also having a multifaceted techno-functional role such as thickening, gelling, foaming and emulsifying properties, among others. Milk proteins are classified as caseins, i.e. αs-, β- and κ-caseins, and whey proteins, i.e. β-lactoglobulin, α-lactalbumin, lactoferrin, immunoglobulins and proteose peptones (Walstra, Walstra, Wouters, & Geurts, 2005). The technological and functional properties of milk proteins can be substantially diversified, due to the conformational dissimilarity of their inherent structure, in which whey proteins exhibit a globular well-defined three-dimensional structure while the casein micelles have a rather loose and more flexible structure (Goulding, Fox, & O’Mahony, 2020).

The exposure of milk protein aqueous dispersions to intragastric ambient conditions (i.e. gastric fluids containing acids, digestive enzymes and counterions) induces remarkable microstructural changes in the proteinaceous food bolus (Ye, 2021). Parameters such as the composition (casein to whey ratio, presence of minerals or lipids) and the structure conformational aspects of milk proteins (native vs denatured, micellar vs colloidal calcium phosphate (CCP) depleted etc.) are inextricably associated with their acid and/or pepsin-induced coagulation, as well as with their proteolytic breakdown throughout gastrointestinal transit (Ye et al., 2020, Ye, 2021). Micellar caseins can undergo extensive coagulation in the presence of milk-clotting enzymes (i.e. pepsin and chymosin) due to the peptic cleavage of the κ-casein eliminating the micelle-stabilising role of *para*-κ-casein, which eventually leads to the formation of protein aggregates (Ye, Cui, Dalgleish, & Singh, 2016). Contrary to micellar casein, the intragastric coagulation of Na- or Ca-caseinate is exclusively mediated via the formation of acid aggregates when intermolecular electrostatic forces repulsion between the CCP depleted casein molecules occurs (Horne, 2020). Whey proteins, i.e. β-lactoglobulin and α-lactalbumin are generally less responsive to intragastric peptic cleavage than caseins, and this is ascribed mainly to the pepsin selectivity for the looser structure of the latter (Dupont & Tomé, 2020). Nevertheless, caseins are classified as “slow proteins”, a term that is used to denote their ability to induce the sustained release of amino acids into the blood plasma as opposed to the acute postprandial amino-acidemic response of whey proteins (Boirie et al., 1997).

In general, common food processing practices such as thermal processing (e.g. pasteurisation, sterilisation, drying etc.) and homogenisation enhance the in-vitro digestibility of milk proteins (Barbé et al., 2013, Böttger et al., 2019, Dupont and Tomé, 2020, van Lieshout et al., 2020). In addition, the chemical modification of milk proteins by means of glycation, oxidation, racemisation, dephosphorylation and enzymatic crosslinking can greatly impact their gastric and intestinal-induced peptic cleavage (van Lieshout et al., 2020). Besides, the heat, acid, rennet or salt mediated *sol*-*gel* physical state transitions that occurs are known for modifying the degree of the proteolytic breakdown of milk proteins during digestion (Barbé et al., 2013, Barbé et al., 2014, Ye et al., 2020). Macierzanka et al. (2012) demonstrated that the pepsinolytic resistance of heat-treated β-lactoglobulin solutions in various pH conditions was significantly lower in the case of fine-stranded protein aggregates formed in either neutral (pH = 6.5) or highly acidic (pH = 2.5) conditions compared to the firm protein coagulates formed at pH ≈ pI (pH = 5.2). Böttger et al. (2019) reported that the gastro-duodenal digestion resistance of the individual casein epitopes (α_s1_-, α_s2_-, β- and κ-) increased when sodium caseinate was crosslinked by means of transglutaminase.

Polysaccharides do not only possess a vital nutritional role as dietary fibres but also confer significant techno-functionalities to food matrices such as structuring, thickening and interface stabilising agents. In general, mixing polysaccharides with milk proteins may result in: a) the co-solubility of the biopolymers, b) associative interactions leading to complex coacervation, and c) a non-associative segregative phase separation due to either thermodynamic incompatibility or depletion flocculation phenomena (Turgeon, Beaulieu, Schmitt, & Sanchez, 2003). Although limitedly studied to date, the types of milk protein – polysaccharide interactions is closely related to the resistance of the milk proteins to the peptic cleavage enzymes (Borreani et al., 2016, David et al., 2020, Koutina et al., 2018, Liu and Kong, 2019, Ma et al., 2021, Markussen et al., 2021). Investigating the impact of neutral (konjac gum) and anionic (sodium alginate) polysaccharides on the in-vitro digestibility of unheated milk proteins Borreani et al. (2016) found that the milk protein pepsinolysis was mainly suppressed by sodium alginate, which was ascribed to the occurrence of electrostatic complexation between sodium alginate and milk proteins. In a consecutive study, Koutina et al. (2018) reported that the molar mass of sodium alginate was adversely related to the in-vitro digestibility of nanoparticulated whey protein, with peptides derived from β-lactoglobulin in the 55–66 and 109–123 regions being the most resistant to peptic cleavage. Markussen et al. (2021) demonstrated that the intragastric release of free amino acids was adversely related to the size of the milk protein concentrate (MPC) – polysaccharide formed coacervates.

In our previous works (Hellebois et al., 2022, Hellebois et al., 2021a), the promising techno-functional aspects of alfalfa (*Medigaco sativa* L.) seed galactomannan were unveiled. The present work reports on the role of alfalfa galactomannan in modulating the in-vitro digestibility of milk protein-based (whey protein isolate and sodium caseinate) liquid food models. In this context, it is hypothesised that alfalfa galactomannan may sustain the peptic cleavage of milk proteins through its ability to control the acid-induced protein aggregation phenomena and hinder sterically the proteases diffusivity at the solid – liquid interface boundaries.

## Materials and methods

### Materials

Whey protein isolate powder (PRODIET 90S®) with a protein content of 85.8% wt. was kindly donated by Ingredia (Arras, France) whereas the sodium caseinate containing 89.4% wt. of protein (N% ×6.25) was purchased from Sigma-Aldrich (Leuven, Belgium). The alfalfa galactomannan (AAG), with a molecular weight of 2.0×10^6^ Da, an intrinsic viscosity of 9.33 dL g^−1^ and a mannose to galactose ratio of 1.18, was isolated and purified following the procedure detailed in Hellebois et al. (2021b). All the other chemicals used were of analytical grade.

### Sample preparation

The appropriate amount of whey protein isolate (WPI) or sodium caseinate (NaCN) was dispersed in Milli-Q to obtain a 10% wt. protein dispersion. The amount of protein content was verified by the Dumas method using an organic elemental analyser (Vario Cube, Elementar GmbH, Langenselbold, Germany). The protein dispersions were kept under magnetic stirring at ambient temperature overnight (IKA GmbH, Staufen, Germany) to allow sufficient hydration followed by centrifugation at 10000*g* for 5 min to remove any insoluble residual. Then, the milk protein solutions were heat treated at 80 ± 1 °C for 20 min in a shaking water bath (Julabo SW22, Seelbach, Germany) before being three times homogenised at 500 bar (Panda plus 2000, Gea, Germany) to break down any protein agglomerates and rapidly cooled down at ambient temperature. The absence of protein agglomerates was confirmed by means of a static laser light scattering particle size analysis (Mastersizer 3000, Malvern Instruments, Worcestershire, UK). To avoid any bacterial growth, 0.02% wt. of sodium azide was added to the heat-treated protein solutions. The milk protein-AAG binary blend solutions were prepared by dispersing the appropriate amount of gum (i.e. 0.1, 0.5 or 1% wt.) into the milk protein solution and allowing the biopolymer to dissolve and fully hydrate under magnetic stirring overnight at ambient temperature. All protein – AAG aqueous systems were degassed by centrifugation (3000*g* for 5 min) prior to their physicochemical and in-vitro digestibility assessment.

### Static simulated in-vitro digestion

In order to conduct the static simulated in-vitro digestion experiments, the INFOGEST v.2.0 protocol was adopted (Brodkorb et al., 2019). Briefly, 10 g of the initial food models (WPI-AAG; NaCN-AAG) were transferred into an Erlenmeyer flask, diluted 1:1 with simulated saliva fluids (SSF) containing α-amylase (150 U mL^−1^), closed with a glass stopper and incubated for 3 min in a shaking water bath at 37 ± 0.1 °C, 100 Hz. Then, the oral bolus was diluted with an equal amount of pre-warmed (to 37 °C) simulated gastric fluids (SGF) containing pepsin (activity in the final chyme of 2000 U mL^−1^) adjusted to pH = 2.5 by adding HCl 1 M (at a rate of 60 μL min^−1^) and incubated for 2 h at 37 °C under constant agitation. The intestine phase was initiated by adding an equal amount of pre-warmed simulated intestine fluids (SIF), containing the bile salts and pancreatin enzyme blend (trypsin activity of 200 U mL^−1^) and adjusted to pH = 7 using NaOH 1 M (at a rate of 60 μL min^−1^) to the gastric chyme. Solution or suspension aliquots were taken from the initial food matrix (FM) both at the end of the oral phase and at pre-selected time intervals (i.e. t = 0, 5, 10, 20, 30, 60, 90 and 120 min) throughout the simulated gastric and intestinal processing steps (120 min total duration for each step). The proteinases activity was stopped by diluting the sample ten-fold in PBS buffer (pH = 7.4) and consecutively adding 10 µL mL^−1^ of protease inhibitor mix (Cytiva, Marlborough, MA, United States). All samples were immediately placed into an ice bath and were analysed.

### In-vitro digestibility assessment

#### Protein digestibility

The protein proteolytic hydrolysis occurring during the in-vitro simulated gastrointestinal transit was tracked by SDS-PAGE analysis as follows: all systems were diluted to 5, 5, 10 and 20 µg per well of proteinaceous matter load for the food matrix, oral bolus, and gastric and intestine chymes, respectively. The samples (7.0 µL) were mixed with 2.5 µL of XT sample buffer (Bio-Rad, Hercules, CA, United States) and 0.5 XT reducing agent (Bio-Rad), heated at 95 °C for 5 min followed by rapid cooling in an ice bath. Aliquots of 10 µL were then transferred into the polyacrylamide (12%) gel wells.

SDS-PAGE electrophoresis was run in XT MES (Bio-Rad) buffer solution at 200 V for approx. 45 min until the protein migration reached the edge of the gel. The proteins were then stained with the Serva Purple fluorescent probe following the manufacturer’s instructions. The imaging of the gel was performed using a Typhoon FLA 9500 Imager (GE Healthcare, Chicago, United States) with a pixel resolution set at 10 µm. The densitometric analysis of the protein molecular weight bands was carried out using the ImageJ software.

#### Protein hydrolysis quantification

The hydrolysis progress of the milk proteins was monitored by quantifying the amount of primary amino groups present in the collected food matrix, and gastric and small intestine chymes using the O-phthaldialdehyde (OPA) method of Halabi, Croguennec, Bouhallab, Dupont, and Deglaire (2020). The food matrix was diluted 50-fold, whilst the gastric and small intestine phases were diluted 25 times. The gastrointestinal phases were then centrifuged for 5 min at 10000*g*, 4 °C and 20 µL of the supernatant were mixed with 150 µL of ready-to-use OPA solution in a UV 96-well microplate (UV-Star® Greiner, Germany) following 2 min of incubation in the dark at room temperature, and the absorbance at 340 nm was measured using a UV/VIS Spark® 20 M microplate reader (Tecan, Männedorf, Switzerland).

To calculate the total amino acid content in the food matrix, acid hydrolysis was performed in each system. One hundred microlitres of the food matrix were diluted to 1 mL with HCl 6 M, and the samples were hydrolysed at 110 °C for 24 h in Pyrex tubes (Hach, Loveland, United States). The hydrolysed samples were neutralised using 1 mL of NaOH 6 M and diluted to 10 mL with deionised water. The free amino acid quantification was then determined as described above. The degree of hydrolysis of the proteins was determined as follows:

(1) $$\text{DH}\left(\%\right)=\frac{{\text{NH}}_{\text{2digested}}-{\text{NH}}_{\text{2FM}}}{{\text{NH}}_{\text{2total}}-{\text{NH}}_{\text{2FM}}}\times \text{100}$$

where DH is the degree of hydrolysis, NH_2FM_, NH_2digested_ denote the primary amino group content of the food matrix and the obtained digesta (gastric or intestine), and NH_2total _is the content of primary amino group in the acid hydrolysed food matrix.

### Confocal laser scanning microscopy (CLSM)

The microstructure of the protein – galactomannan biopolymer systems was characterised by means of CLSM imaging mounted with a ×10 objective lens. In brief, 1 mL of the initial food matrix, and oral, initial and end gastric chyme, as well as the initial and end small intestine digesta were rapidly transferred into 1 mL Eppendorf tubes, mixed with 10 µL of protease inhibitor to cease the in-vitro digestion process and were stored in an ice bath until microscopic evaluation. The proteins were non-covalently stained using 10 µL mL^−1^ of Fast Green 0.05% wt. aqueous solution. To assess the protein microstructure, aliquots of 300 µL were transferred into eight-well Nunc Lab-Tek® II chamber microscope slides. The excitation and signal emission wavelengths used were 633 and 635–735 nm, respectively.

### Rheological analysis

The steady-state flow behaviour of the individual proteins and protein-AAG solution was measured using an oscillatory rheometer (MCR 302, Anton Paar, Graz, Austria). A double-gap (DG 26.7) geometry of 27.1 mm diameter was used to characterise the flow behaviour of the food matrix, buccal, gastric and intestinal samples (at 37 °C) at shear rates ranging from 1 to 100 s^−1^. For comparison reasons, the consistency coefficient values (K, in Pa.s*^n^*) of the orogastrointestinal phases were determined by fitting the shear stress – shear rate data to either pseudoplastic (Ostwald – de Waele) or Newtonian flow behaviour models. To assess the contributory effect of the insoluble acid/pepsin induced protein aggregates to the viscosity, the gastric chymes were centrifuged for 5 min at 4500*g* and the obtained supernatants were also characterised rheologically as aforementioned.

### Statistical analyses

The Shapiro-Wilk test and Q – Q plot representation normality tests were used to verify the normal distribution of the data. To determine the significance of AAG addition on the in-vitro digestibility of the protein, one-way ANOVA was performed using Origin 2019b software (OriginLab Inc, USA). Tukey’s multiple range test was used to separate mean values when significant differences (p < 0.05) were detected.

## Results and discussion

### Characterisation of the model food matrices and boluses

In the absence of AAG, both milk protein solutions exhibited a Newtonian flow behaviour with the NaCN, exhibiting generally higher apparent viscosities than the WPI exemplars (Suppl. Fig. 1A and B). As expected, the presence of AAG in the milk protein solutions resulted in a well-defined shear thinning flow behaviour (Hellebois, Gaiani, & Soukoulis, 2022) with their apparent viscosities increasing proportionally to the AAG concentration (c_AAG_). In general, at c_AAG_ ≥ 0.5% wt., the changes in the apparent viscosities of the milk protein solutions became more pronounced (Suppl. Fig. 1). It is well-documented that the rheological behaviour of galactomannan solutions is directly related to their structure conformational properties such as the mannose to galactose (M/G) ratio, the sequence of the galactose depleted (smooth) to galactose substituted (hairy) regions, the molar mass and hydrodynamic radius (Doyle et al., 2009, Hellebois et al., 2021a, Sittikijyothin et al., 2005). As for alfalfa galactomannan, we have previously shown that at c_AAG_ = c* ≈ 0.3% wt. the interchain polymer-polymer interactions became significant, resulting in a tangible increase in their apparent viscosity values (Hellebois et al., 2021a). Besides the direct contribution of the AAG to the flow behaviour characteristics of the WPI solutions, the occurrence of segregative microphase separation phenomena in biopolymer binary blends are also known to induce synergistic or antagonistic effects on viscosity owing to the mutual exclusion of each polymeric component in the microdomain of the other one (Schorsch et al., 2000, Sittikijyothin et al., 2010). In the case of WPI-AAG binary blends, a segregative microphase separation at c_AAG_ ≥ 0.1% wt. driven by a depletion-flocculation mechanism was reported (Hellebois et al., 2022). According to the CLSM micrographs acquired (Fig. 2; Suppl. Fig. 2), all binary WPI-AAG systems were microphase-separated, exhibiting diversified structure conformational aspects, i.e. AAG-rich microdroplets constrained into the continuous WPI-rich aqueous phase (c_AAG_ = 0.1% wt.), bicontinuous (c_AAG_ = 0.5% wt.) or protein aggregated-like (c_AAG_ = 1% wt.) microstructures. Contrary to WPI-AAG solutions in which macroscopically (visually detected) separated phases could be detected for all tested protein – galactomannan concentrations, in the case of NaCN-AAG exemplars, it was very difficult to visually detect the occurrence of phase separation. The CLSM-assisted assessment of the microstructural features of the NaCN-AAG confirmed the demixing of the biopolymers at the microscopic scale, which is in agreement with the observations of López, Galante, Alvarez, Risso, and Boeris (2017) on NaCN – *Gleditsia amorphoides* galactomannan systems. Although the proteins in NaCN exert a dissociated non-micellar structure conformation, dynamic light scattering studies have evidenced the ability of sodium caseinate to undergo self-assembly, resulting in the formation of rod-like polymer associations that are in accordance with the adhesive hard sphere model (Farrer & Lips, 1999). Also, taking into account the negligible surface charge density of AAG (Suppl. Fig. 3), which renders the electrostatic (repulsive) interactions between AAG and NaCN rather insignificant, it can be postulated that the demixing phenomena in the NaCN-AAG systems are due to depletion – flocculation.

**Fig. 1:**
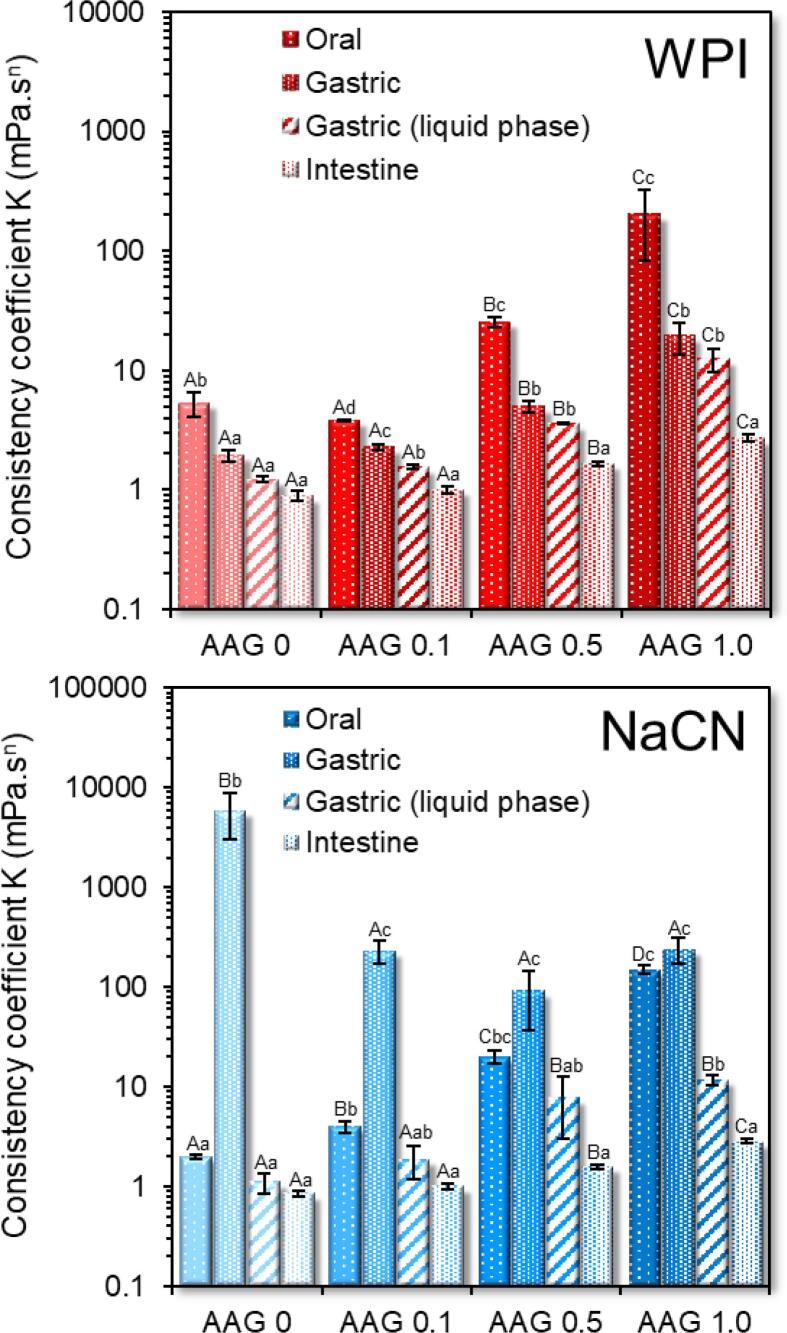
Consistency coefficient K of the buccal, gastric and intestinal phases obtained as influenced by the presence of alfalfa galactomannan. All systems were measured at 37 °C from 1 to 100 s^−1^. For comparison purposes, the consistency coefficient of the gastric chymes with or without protein aggregates (centrifuged at 4500*g* for 5 min) are reported. ^A−D, a−e^Different letters between the bars denote a significant difference among samples differing in the amount of AAG (uppercase) or in the in-vitro digestion step (lowercase).

**Fig. 2:**
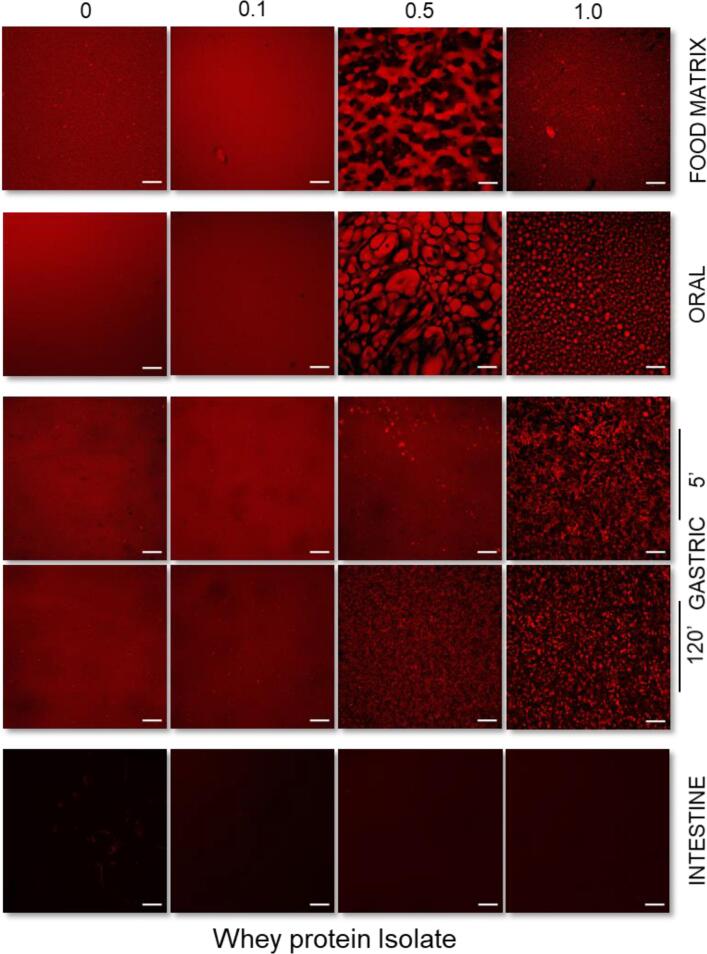

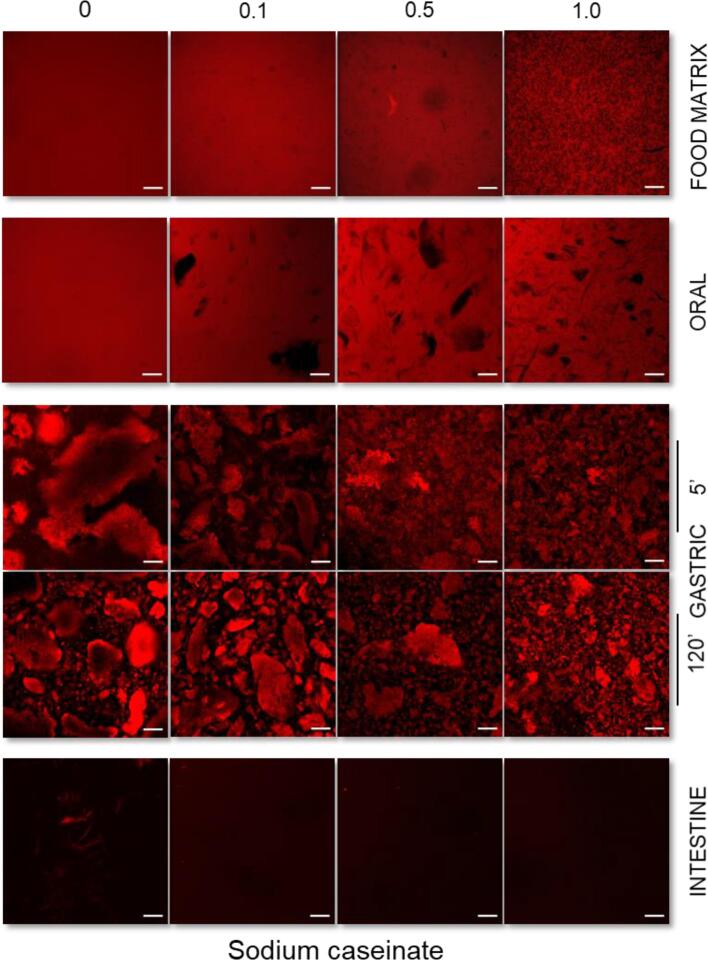
Confocal laser scanning microscopy acquired micrographs of the initial protein-based models and the buccal, gastric, and intestinal phases obtained as influenced by the milk protein type (WPI vs NaCN) and the amount of alfalfa galactomannan (0.1, 0.5 or 1% wt.). Scale bar = 100 μm.

On admixing the milk protein food models with the SSF, an abrupt decrease in the consistency coefficient values of the obtained food boluses was observed, as expected (data not shown). Significant microstructural changes may come about in the formation of the bolus in liquid protein – polysaccharide systems, which are primarily associated with the modification of the homopolymer and heteropolymer interactions due to the transition from the semi-dilute to the dilute state, as well as the increase in the ionic strength (Soltanahmadi, Murray, & Sarkar, 2022). According to the CLSM micrographs (Fig. 2), evident microstructure conformation changes were observed, i.e. micro-aggregated to water-in-water emulsion-like, and interconnected to bicontinuous phase-separated structures. On the other hand, AAG-rich microdomains distributed in the protein-rich bulk (continuous) aqueous phase were detected in the case of the NaCN-based buccal phases.

**Fig. 3:**
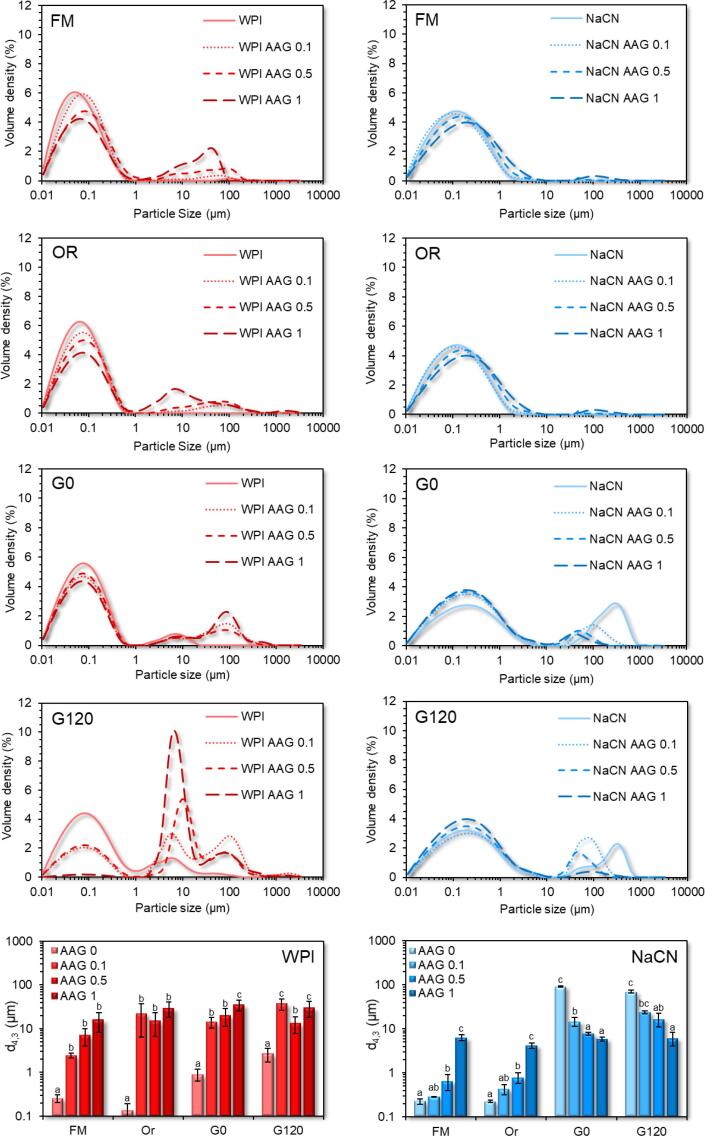
Particle size distribution curves and d_4,3_ values (in μm) of the WPI (red lines) and NaCN (blue lines)-based food models (FM) and the in-vitro oral (OR) and gastric phases obtained at t = 0 (G0) and t = 120 min (G120) as influenced by the AAG content (0.1 – 1% wt.). ^a−c^Different letters between the bars denote a significant difference among samples differing in the amount of AAG. (For interpretation of the references to colour in this figure legend, the reader is referred to the web version of this article.)

Static light-scattering measurements of the food matrices and the boluses obtained (Fig. 3) confirmed the presence of soluble oligomeric aggregates in WPI (ca. 260 nm) and NaCN (ca. 220 nm) dispersions. The presence of AAG was associated with incremental changes in the mean size of the WPI (0.140 to 29.35 μm) and NaCN (0.285 to 6.830 μm) dispersions. The changes observed can be primarily ascribed to excluded volume effects, i.e. as the opposite polymer-rich microdomains become more concentrated, the alike polymer-polymer interactions are favoured, leading to the formation of loosely associated (via hydrogen bond or hydrophobic interactions) soluble aggregates (Hellebois et al., 2022).

### In-vitro gastrointestinal digestion

The oral boluses were sequentially exposed to in-vitro gastric and small intestine conditions in order to monitor the colloidal changes and digestibility of the proteins influenced by the presence of AAG. The sampling of the gastric and intestinal chymes at pre-determined time intervals aimed to provide a simplistic overview (owing to the *per se* technical limitations of the static models compared to the semi-dynamic and dynamic models in order to accurately simulate the physiological GI conditions) of the dynamics of the acid-induced aggregation, matrix disintegration and proteolysis. Due to the protein/polysaccharide buffering effects, the amount of HCl added to the gastric chymes was customised for each protein food model. The sampling of the gastric chymes commenced (t = 0 min) when the target pH of 2.5 was achieved, to ensure maximal pepsin activity (Kondjoyan, Daudin, & Santé-Lhoutellier, 2015).

#### Microstructural changes

The macroscopic (visual) evaluation of the gastric chymes obtained at t = 5 min (after achieving pH = 2.5) unveiled the formation of large aggregates undergoing fast sedimentation under quiescent conditions (NaCN) or smaller protein clots resulting in a more uniform suspension (WPI). In the case of NaCN, the presence of AAG was related to a reciprocal to galactomannan concentration reduction in the mean size of the protein aggregates, resulting in a thickened, less heterogeneous suspension. The macroscopic observations were also confirmed on a microscopical scale as illustrated in the acquired CLSM micrographs (Fig. 2) and the SLS measurements (Fig. 3). In the latter case, all systems exhibited a bimodal particle size distribution with the d_4,3_ values (ranging from 5.8 to 92 μm) adversely correlated (p < 0.05) to the c_AAG_ (Fig. 3). The d_4,3_ values reported here are generally in the range of those reported in the literature (Borreani et al., 2016, Markussen et al., 2021). Depending on their chemical structure, polysaccharides may affect the colloidal aspects of milk proteins when exposed to gastric fluids via different mechanistic pathways, including electrostatic complexation, microscopic phase separation or the thickening of the continuous phase (Borreani et al., 2016, Koutina et al., 2018, Markussen et al., 2021). To assess the AAG contribution to the macroviscosity of the continuous phase, the consistency coefficient of the gastric chymes before and after centrifugation was determined (Fig. 1). As expected, a strong positive correlation between d_4,3_ and consistency coefficient values (r = 0.921, p < 0.001) was found, which is in accordance with the findings of Markussen et al. (2021) for guar gum-milk protein concentrate systems. Following centrifugation, a steep reduction in the consistency coefficient values of the continuous phase was observed, approaching those of the pure AAG solutions (Hellebois et al., 2021b). Nevertheless, minor deviations due to the pH, temperature and ionic strength in the hydrodynamic volume of AAG are expected (Repin, Cui, & Goff, 2018). In this context, it appears that the SGF-induced colloidal transformation of the NaCN-AAG boluses stems from the thermodynamic incompatibility of the biopolymers (Markussen et al., 2021) and the ability of AAG to slow down the migration of pepsin and acid to the solid-water interface (Borreani et al., 2016).

In the case of the WPI-based gastric chymes, it was not possible to note any distinct visual changes in the size of the acid formed aggregates, with all systems to exhibit a quite homogeneous appearance. On this occasion, CLSM analysis was found to be more assistive in evaluating the morphological differences of the protein aggregates formed (Fig. 2). Contrary to NaCN-based gastric chymes, an increase in the size of the protein aggregates (ranging from ca. 0.9 to 35 μm) as a function of c_AAG_ was verified by means of CLSM and SLS measurements (Fig. 2, Fig. 3). As pepsin has a negligible effect on whey protein clotting (Dupont & Tomé, 2020), the aggregates were formed mainly due to protein interchain bridging via nonspecific (e.g. electrostatic, hydrogen bond and hydrophobic) interactions. According to the WPI-AAG phase diagram (Hellebois et al., 2022), and without taking into consideration the possible contributory effects of the electrolytes, it is expected that extensive segregative phase separation will occur at c_AAG_ > 0.5% wt. for the initial food models (0.125% and 2.5% wt. for AAG and WPI, respectively in the gastric chyme). The gastric chymes obtained from the WPI food models containing 0.5% wt. AAG appeared to be very close to the binodal curve (i.e. the boundary between mono- and biphasic protein-polysaccharide systems). Hence, it can be postulated that the gastric-induced aggregation of WPI-AAG is primarily driven by the thermodynamic incompatibility of the two biopolymers (Markussen et al., 2021).

Following 2 h of in-vitro gastric processing, a re-organisation of the microstructural features of the protein aggregates was generally observed (Fig. 2). Nevertheless, the polydisperse character of the particle size distributions was maintained, without any significant reduction in the recurrence of large particle populations being observed for either protein type (Fig. 3). This was also reflected in the d_4,3_ values measured, which was attributed to the inadequacy of the static in-vitro digestion model to accurately reproduce the conditions leading to the formation of protein aggregates (the d_4,3_ values reported here are the average of at least six independently produced gastric chymes). Furthermore, a tangible reduction in the d_4,3_ values of NaCN gastric chymes from 92 to 70 μm was found. Although CLSM micrographs provided some evidence of particle disintegration, the d_4,3_ values for the NaCN containing AAG remained rather unaltered (i.e. ranging from 6.2 to 24.1 μm), most probably due to the enrichment of the above-micron particle populations with fragmented aggregates derived from the very large protein particles (d_90_ = 50 – 160 μm).

With the exception of the WPI-AAG 1% wt. chymes, where to some extent the gastric processing assisted in eroding the particulates formed initially (Fig. 2, Fig. 3) as the concomitant result of the proteolytic attack and mechanical forces, the CLSM micrographs of the remaining gastric chymes were comprised of slightly larger particles. Yet the changes in the d_4,3_ values were not significant, accounting for 0.9, 15.4 and 19.9 µm at the beginning of the gastric digestion to 2.1, 27.6 and 13.8 µm at the end for the NaCN control, 0.1% and 0.5% wt. AAG. It is assumed that these changes may be associated with the ripening of the protein particulates during the early stages of the gastric processing. A similar pattern has been reported outlining the evolution of the d_4,3_ values in MPC-polysaccharide gastric phases (Markussen et al., 2021).

Admixing the gastric chymes with SIF resulted in an abrupt reduction in the consistency coefficient values (Fig. 1), which was attributed to the almost spontaneous disintegration of the protein particles (Fig. 2). Due to limitations associated with the very low mean size of the protein particles (<500 nm) and extensive dilution, it was not possible to get a reliable overview of the microstructural aspects of the intestinal phases by means of CLSM and SLS. Even though the consistency coefficient of the intestinal chymes increased proportionally to the AAG content, no dependence on the protein source i.e., WPI vs NaCN was identified. Similarly to the centrifuged gastric chymes, the luminal viscosities were very close to those previously reported for pure AAG aqueous dilute solutions (Hellebois et al., 2021b) and thus, it can be assumed that AAG maintains its structure conformational properties and molecular intactness throughout gastrointestinal transit.

#### Protein digestion

**Fig. 4:**
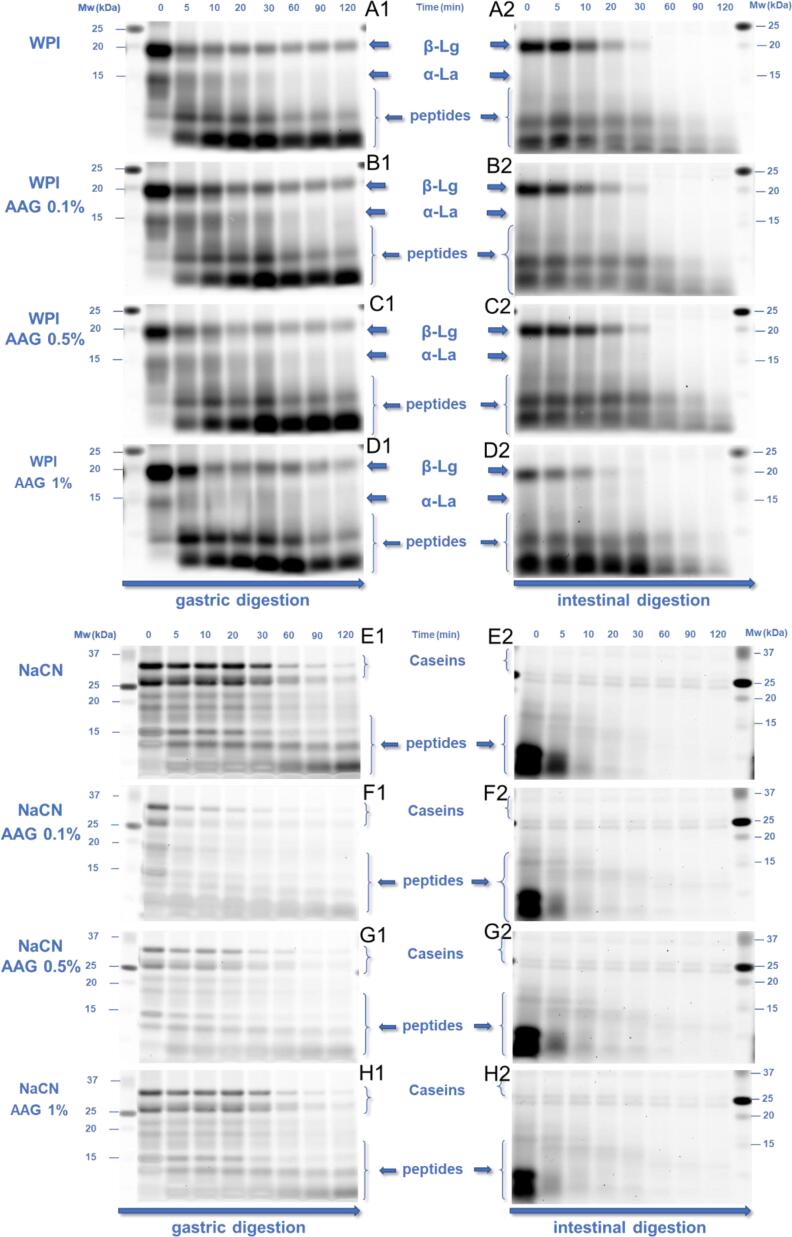
SDS-PAGE patterns of the gastric (1) and intestinal (2) digesta of the WPI (A-D) and NaCN (E-H)-based food models as a function of gastric and intestinal digestion time (0, 5, 10, 20, 30, 60, 90 and 120 min) as influenced by the AAG content (0.1 – 1% wt.).

**Fig. 5:**
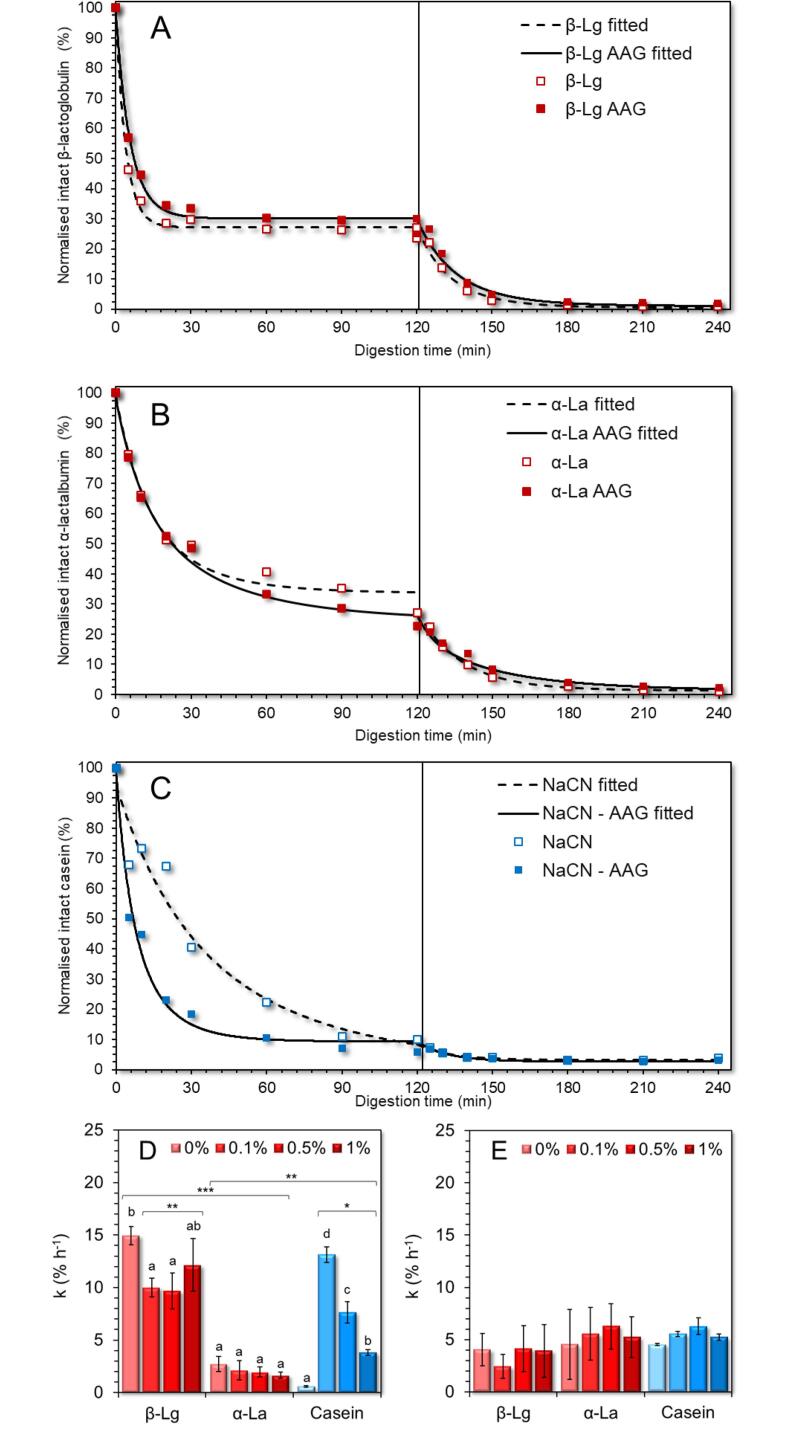
SDS-PAGE densitometric analysis of the protein fate of β-lactoglobulin (A), α-lactalbumin (B) and total caseins (C) during gastric (t_0_ – t_120_) and intestinal (t_120_ – t_240_) simulated static *in-vitro* digestion with or without AAG. D and E display the protein cleavage rate in the gastric and intestinal digesta, respectively. ^a−d^Different letters between the bars denote a significant difference among samples differing in the amount of AAG.

The gastrointestinal fate of the proteins was evaluated by monitoring the release of free amino acids and the amount of residual intact proteins. Since different protein concentrations were used to improve the detection of the intact proteins and their fragments in the buccal, gastric and intestinal phases (i.e. 5, 10 and 20 μg of proteinaceous matter per well), the comparison of the SDS-PAGE images (Fig. 4) was made separately for each in-vitro digestion stage. Concerning the oral boluses, the proteolytic activity observed was negligible, most probably due to the absence of proteases in the artificial saliva (data not shown). In keeping with the literature, caseins (α_s_-, β-, and κ-) were more susceptible to peptic cleavage in the gastric phases than WPI (Ye et al., 2020). It is well established that whey proteins, i.e. β-lactoglobulin and α-lactalbumin, possess a high pepsinolytic resistance, which is primarily associated with their native compact structure conformation (Huppertz & Chia, 2021). The SDS-PAGE were image-processed using ImageJ software to obtain a representative semi-quantitative estimation of the residual matter of the principal intact proteins, i.e. total (α_s_−, β−, and κ^−^) caseins (at 25–35 kDa) for the NaCN and β-lactoglobulin (at 18.2 kDa) and α-lactalbumin (at 14.4 kDa) found in the NaCN and WPI-based digesta. The SDS-PAGE densitometric data were fitted to an empirical, fractional conversion model that showed the best data fitting (Verkempinck et al., 2022) as follows:

(2) $$\text{c}\;={\;\text{c}}_{\text{120}}+\;\left({\text{c}}_{\text{0}}-{\text{c}}_{\text{120}}\right){\text{e}}^{-\text{kt}}$$

(3) $$\tau \;=\;\frac{\text{ln2}}{\text{k}}$$

where c_0_, c_120_ denote the % of intact protein content at the beginning and end of the gastric or intestinal step, and k (% h^−1^) is the rate of the peptic cleavage of proteins to polypeptides and τ is the time required to achieve a 50% reduction in the amount of intact proteins during the gastric or intestine digestion steps. As seen in Fig. 5, the peptic cleavage of proteins was more extensive in the gastric chymes than in the intestinal digesta. Under in-vitro gastric conditions, approximately 72, 2.8 and 15.2 min were required to achieve a 50% reduction in the amount of intact total caseins, β-lactoglobulin and α-lactalbumin, respectively. At the end of the in-vitro gastric processing, the residual intact protein matter (normalised to t = 0 min) accounted for 10.1, 24.0, and 27.0% in the case of total caseins, β-lactoglobulin and α-lactalbumin, respectively. These values are generally in accordance with previous studies, confirming that caseins are more susceptible to pepsinolysis than whey proteins (Halabi et al., 2020, Phosanam et al., 2021). Even though native whey proteins are generally recognised for their substantially higher intragastric peptic resistance (Borreani et al., 2016), the implementation of thermal processing is known as impacting proportionally to its severity the pepsinolytic breakdown of whey proteins (Barbé et al., 2014, Macierzanka et al., 2012, Peram et al., 2013). The latter explains the relatively low amount of intact whey proteins detected in the gastric chymes. The addition of AAG induced a significant (p < 0.05) increase in the pepsin cleavage rates of total caseins (τ = 72.1 and 6.5 min for control and AAG-containing samples) in the NaCN-based food models. This is most probably associated with the ability of AAG to reduce the average size of the protein aggregates and therefore, to facilitate the access of the pepsin to the cleavage sites on the protein molecules. However, among the AAG containing chymes the severity of pepsinolysis was adversely associated with the c_AAG_ (a reduction in the k_gastric_ values from 0.23 to 0.07 % min^−1^ was observed), implying that the thickening effect of AAG may affect the migration rate of pepsin to the solid-water interfaces. In the case of WPI-based food models, only β-lactoglobulin was significantly affected by the presence of AAG (τ = 4 min, p < 0.01); however, no clear correlations between the rate of the peptic cleavage and c_AAG_ were found.

The mixing of the gastric chymes with SIF was accompanied by an abrupt decrease of the total caseins, β-lactoglobulin and α-lactalbumin molecular bands (Fig. 4). According to the densitometric data, it was found that k_intestinal_ < k_gastric_, which can be ascribed to the fact that SDS-PAGE dictates only the cleavage of proteins to oligopeptides and not the extent of peptidolysis nor the amount of free amino acids released. Interestingly, no significant differences in the k_intestinal_ values could be detected for any of the protein species, which indicates that the protease- and peptidase-induced cleavage of the proteins is rapid and less selective than the pepsin-induced proteolysis.

**Fig. 6:**
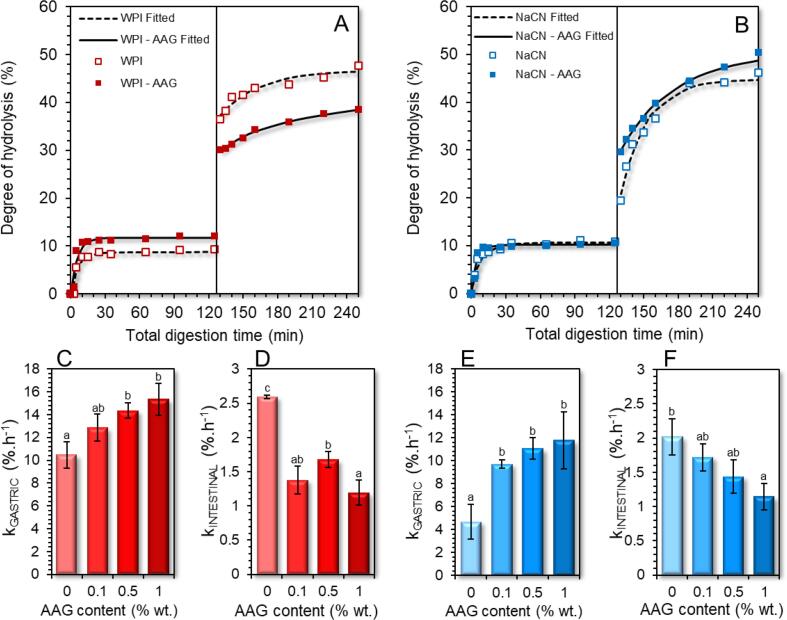
Degree of protein hydrolysis of whey protein isolate (A) and sodium caseinate (B) during gastric and intestinal *in-vitro* digestion. The protein hydrolysis rates (k) of WPI and NaCN during gastric and intestinal steps are displayed in C-F. ^a−c^Different letters between the bars denote a significant difference among samples differing in the amount of AAG.

To gain a more complete insight into the degree of protein hydrolysis, i.e. the cleavage of peptides and oligopeptides to free amino acids, the gastric and intestine chymes were analysed by means of OPA assay and the data obtained were fitted to the following first-order kinetics model (Le Feunteun et al., 2021):

(4) $$\text{DH}={\text{DH}}_{\infty }\left(\text{1}-{\text{e}}^{-\text{kt}}\right)$$

where DH_∞_ denote the degree of hydrolysis (%) at end of the gastric or intestinal digestion step and k (% h^−1^) is the rate of peptic cleavage of proteins to polypeptides. In general, the DH of all protein food models (with or without AAG) did not exceed 12% under intragastric conditions (Fig. 6A and B), with most of the observed proteolytic changes occurring in the very early stages (τ = 3 to 8 min) of gastric processing. The limited DH of milk proteins during the gastric processing stage has been also confirmed in other studies (Halabi et al., 2020, Liu and Kong, 2019, Rinaldi et al., 2014). According to the calculated k_DH_ values (Fig. 6C and E), the presence of AAG was associated with a significant (p < 0.05) increase in the release rates of free amino acids, accounting for 22–47% and 107–152% for NaCN and WPI-based food models, respectively. These findings imply that under the conditions tested here, the release kinetics of free amino acids come mainly from oligomeric protein aggregates and therefore, the ability of AAG to mediate the acid-induced ripening of the protein aggregates in the very early-stage gastric processing.

As regards the intestinal digestion stage, the DH increased rapidly within the first 5 min of exposure to the intestinal proteases and peptidases, followed by a progressive increase in the amount of the free amino acids released until they reached a final DH of 36 to 52%, which is in line with the values reported for NaCN and WPI. The kinetic modelling of the DH data (Fig. 6D and F) revealed that the presence of AAG suppressed the k_intestinal_ values. Nevertheless, a reciprocal interrelationship between c_AAG_ and k_intestinal_ values was only observed in the case of NaCN-based digesta.

## Conclusions

The aim of the present study was to investigate the effect of an underexplored galactomannan isolated from alfalfa seeds on the in-vitro digestibility of milk proteins. Although the addition of AAG improved the rheological profile of the initial milk protein-based liquid food models, it also induced extensive segregative phase separation phenomena driven via a depletion flocculation mechanism. The food boluses retained their phase-separated microstructure, but noticeable structure conformational differences were detected depending on the protein type and AAG content. Extensive protein aggregation phenomena in the obtained gastric chymes were confirmed both by CLSM and static light-scattering analysis. The addition of AAG had a modulatory role in controlling the average size of the acid-induced protein particulates and therefore their proteolytic resistance against pepsin-induced cleavage. The increase of the AAG content in NaCN gastric and intestinal chymes favoured the proteolytic attack of the protein aggregates formed. On the other hand, no clear correlations between the AAG concentration and the degree of proteolysis were found; however, the presence of AAG-facilitated pepsin induced the cleavage of β-lactoglobulin. In this context, the present study confirmed that alfalfa galactomannan can be successfully implemented not only as an efficient thickening/structuring agent in dairy based food systems, but it can also be exploited as a non-digestible biopolymer to tailor their post-ingestion colloidal and bio-functional performance. Its role as co-structuring biopolymer in milk protein-based delivery carriers will be showcased in a future study.

## Declaration of Competing Interest

The authors declare that they have no known competing financial interests or personal relationships that could have appeared to influence the work reported in this paper.
