# Supplementary material for: Impact of alfalfa (Medicago sativa L.) galactomannan on the microstructural and physicochemical changes of milk proteins under static in-vitro digestion conditions
Source: Food Chem X. 2022 May 18;14:100330. doi: 10.1016/j.fochx.2022.100330 (PMC9125660; doi:10.1016/j.fochx.2022.100330)
Supplement: Supplementary data 1 [file mmc1.docx]

SUPPLEMENTARY MATERIAL


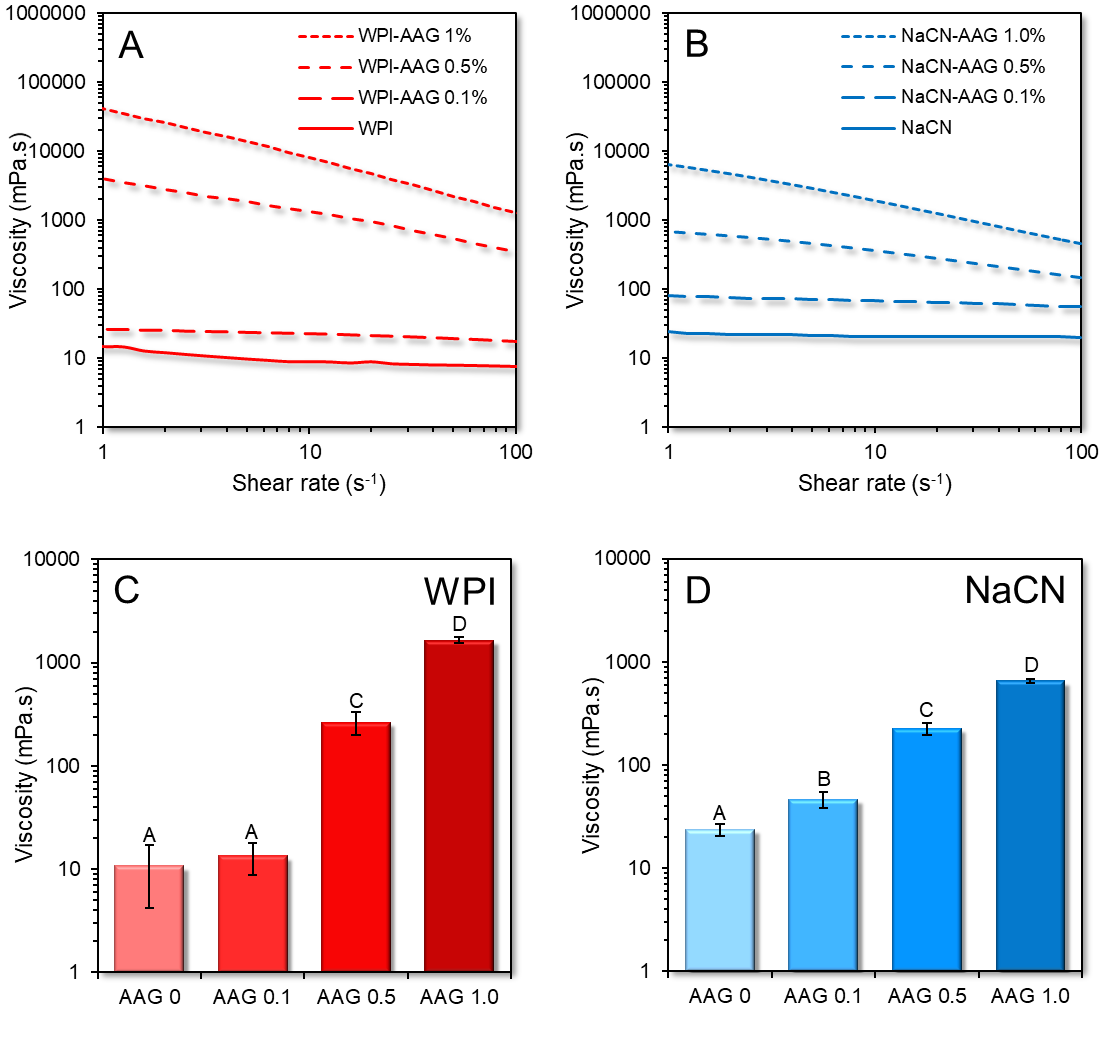


SUPPLEMENTARY FIGURE 1: Flow behaviour curves of WPI (A) and NaCN (B) solutions and their respective viscosity recorded at 50 s^-1^ (C,D) as influenced by the presence of AAG (0.1-1% wt.). ^A-D^Different letters between the bars denote a significant difference among samples differing in the amount of AAG.


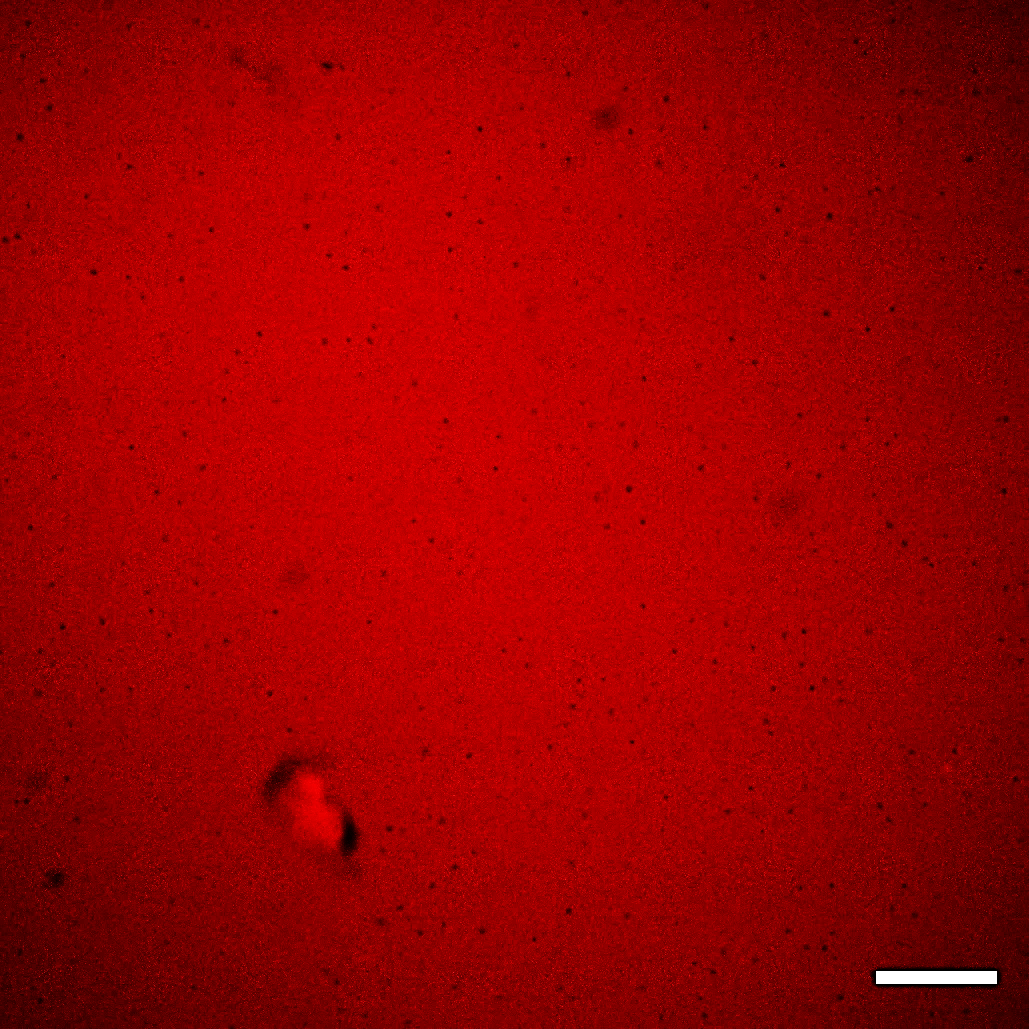


SUPPLEMENTARY FIGURE 2: Confocal laser scattering microscopy acquired micrograph of the WPI-AAG 0.1% wt food matrix highlighting each water-in-water like microstructural conformation. Scale bar = 100 μm


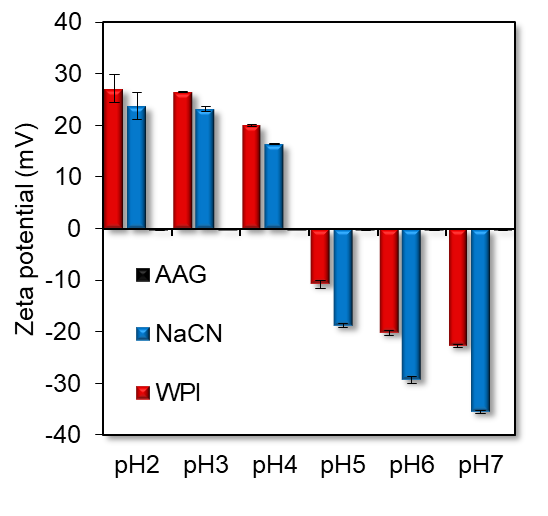


SUPPLEMENTARY FIGURE 2: Surface charge density (ζ-potential) of individual biopolymer systems i.e., NaCN, WPI or AAG as influenced by the pH. The surface charge density of individual WPI, NaCN and AAG solutions (0.1% wt.) was assessed using dynamic light scattering (Zetasizer Nano ZS, Malvern Instruments Ltd, Worcestershire, UK). Refractive indices of 1.450, 1.341 and 1.334 for WPI, NaCN and AAG, respectively, were adopted.
